# Lifting the veil on fungal toxins

**DOI:** 10.1038/cddiscovery.2016.35

**Published:** 2016-05-30

**Authors:** S Pasricha, J S Pearson

**Affiliations:** 1 Department of Microbiology and Immunology, University of Melbourne at the Peter Doherty Institute for Infection and Immunity, Melbourne 3000, Australia

The outcome of a pathogenic microbial infection is dictated by host−pathogen interactions and requires constant rapid adaptive responses by both the microbe and the host. While pathogenic bacteria and viruses promote infection via the ‘proactive’ secretion of toxins and virulence proteins to hijack host machinery, the dogma of fungal pathogenesis to date has been one of almost ‘accidental’ evolutionary, adaptive survival. For many fungal pathogens, pathogenesis begins with adapting from a saprophytic/mutualistic to a parasitic lifestyle within the host. Numerous studies have focussed on how fungal pathogens defensively subvert exposure to mammalian host immune response-induced stresses and overcome nutritional restrictions.

*Candida albicans* is a fungal commensal of human skin and mucosal surfaces that can transition into an invasive fungal pathogen within immunocompromised individuals. Annually, *C. albicans* infection results in over 400 000 cases of invasive disease worldwide and systemic infection leads to high mortality rates.^1,2^ Like many opportunistic fungi, a key virulence factor of *C. albicans* is the ability to undergo a reversible morphological switch from a unicellular (yeast) to a filamentous (hyphal or pseudohyphal) growth form. This switch, resulting in changes of both cell shape and cell physiology, is thought to allow fungal pathogens to adapt to different environmental conditions and has been correlated with pathogenicity traits.^3–5^

*C. albicans* normally remains benign on the mucosal surfaces of healthy humans. When its host becomes immunocompromised *C. albicans* can proliferate, disseminate and form invasive filamentous hyphae. Upon uptake by host macrophages, it is believed that hyphal tip extension by *C. albicans* produces enough pressure to penetrate and pierce through the macrophage cell wall.^6,7^ Hyphal-specific adhesion proteins have also been shown to bind to host epithelial cells. This leads to the induction of endocytosis, facilitating hyphal invasion of host epithelial cells.^8^ While it is clear that signals within macrophages, for example, can induce the dramatic morphological transition to hyphae, the molecular mechanisms behind the pathogenic potential of hyphal cells have remained unclear.^9^

In a paper recently published in *Nature*, Moyes *et al.*^10^ screened a library of *C. albicans* gene deletion mutants for their ability to cause damage to oral epithelial cells and to trigger host immune signaling pathways. Deletion of the extent of cell elongation 1 gene (*ECE1*) resulted in normal transition from yeast to hyphal cells during infection, but led to the inability of hyphal cells to cause epithelial damage and prevented p-MKP1/c-Fos-mediated danger responses and cytokine secretion *in vitro* or in two *in vivo* mucosal models: zebrafish swimbladders and mouse models of thrush. This finding highlighted the important role of Ece1p, from the perspective of both the pathogen and the host.

For almost two decades the role of *ECE1* has remained elusive. *ECE1* was found to be highly upregulated in *C. albicans* hyphal cells compared to yeast cells and resulted in the biosynthesis of a large polypeptide. The function of this polypeptide, however, was unknown and the deletion of *ECE1* did not prevent the transition from yeast to hyphal cells. Through the use of recent advanced technologies, Moyes *et al.* showed that Ece1p is in fact cleaved at 7-lysine-arginie motifs into eight shorter peptides by *C. albicans* protease Kex2p. All eight peptides were secreted by hyphal cells *in vitro*. Individually incubating each peptide with epithelial cells revealed that only one cleaved product, the Ece1-III peptide, caused epithelial damage and induced danger responses in human epithelial cells. Cementing the role of the Ece1-III peptide further, a *C. albicans* strain lacking only the genome region encoding the Ece1-III peptide was shown to grow as morphologically normal hyphal cells, but was unable to cause damage to epithelia, induce p-MKP1/c-Fos-mediated danger responses or cause mucosal disease in mouse animal models. This phenotype was consistent with an *ece1*Δ/Δ null mutant.

Structural analysis of the Ece1-III peptide suggested that this secreted peptide shares multiple features with that of other cytolytic toxic peptides, such as melittin (honey bee). Characteristically, the Ece1-III peptide is an amphipathic protein containing an amino-terminal *α*-helical hydrophobic region. The peptide binds and permeabilizes lipid bilayers, a process that was enhanced in the presence of cholesterol.

The authors conclude by stating that the Ece1-III peptide is the first identified cytolytic peptide toxin secreted by a human fungal pathogen during infection, naming it Candidalysin. Candidalysin causes lesions to the host epithelial membrane, reminiscent of bacterial cytolytic toxins. This finding changes the dogma in the field of dimorphic fungal pathogenesis from one in which environmental cues trigger an almost ‘accidental’ physiological switch for survival to a switch that is far more parasitic and ‘active’ in nature. Principal questions now present themselves: Do fungal pathogens utilise more similar virulence strategies to pathogenic bacteria than we thought? And is the cleavage of polypeptides a universal strategy of bacterial and fungal pathogens that needs to now be critically assessed?

Moving forward, the research by Moyes *et al.* has opened up many exciting avenues of research to pursue, as well as many pressing questions. In the past, investigation of cell death pathways has mainly taken place in the context of bacterial or viral infection. More recently a number of studies have shown that *C. albicans* triggers NLRP3 and NLRP4-mediated pyroptosis in macrophages.^11,12^ Pyroptosis is thought to occur early in infection (within 8 h), whereas at later stages of infection infected macrophages undergo pyroptosis-independent cell death that requires robust hyphal morphogenesis.^13^ Given this, it may be possible that Candidalysin plays a role in triggering these cell death mechanisms, and possibly other unexplored mechanisms such as necroptosis (Figure 1).

Inflammasome activation can be triggered in the host cytosol by various microbial products including lipopolysaccharide, dsDNA, dsRNA, flagellin, muramyldipeptide, and bacterial toxins such as anthrax lethal toxin.^14^ Therefore it is feasible that Candidalysin may directly activate inflammasome signaling during *C. albicans* infection. As for necroptosis, many pathways can lead to activation of this caspase-independent form of programmed cell death, including stimulation of TNFR1, Fas, TLR3, and TLR4, with the common mediator being RIPK3.^15^ Given that mechanisms of necroptosis are still heavily under investigation, especially those mediated by the DNA-dependent activator of IFN-regulatory factors, it will be interesting to determine whether Candidalysin triggers a RIPK3-dependent necroptosis pathway. Alternatively, like some bacterial virulence proteins, Candidalysin could potentially target immune signaling mediators for inactivation or degradation and thus subvert the cell’s ability to respond adequately to infection, reducing inflammatory cytokine production and increasing growth of the pathogen. An increase in hyphal morphogenesis may promote pyroptotic cell death and other death mechanisms, followed by an increase in the release of danger-associated molecular patterns.

Recent years have seen a threatening increase in the prevalence of opportunistic fungal pathogens. This is believed to be due to the rising numbers of immunocompromised patients, and individuals receiving organ transplants and cancer therapies. Understanding both the selective pressures and mechanisms employed by the different cell types of polymorphic fungi has been fundamental to deducing possible virulence factors and developing infection control strategies. The identification of secreted toxins such as Candidalysin will hopefully provide novel therapeutic targets and open fresh investigations into mammalian immune responses that are essential in fighting fungal infections, particularly inflammatory cell death mechanisms such as pyroptosis and necroptosis.

## Figures and Tables

**Figure 1:**
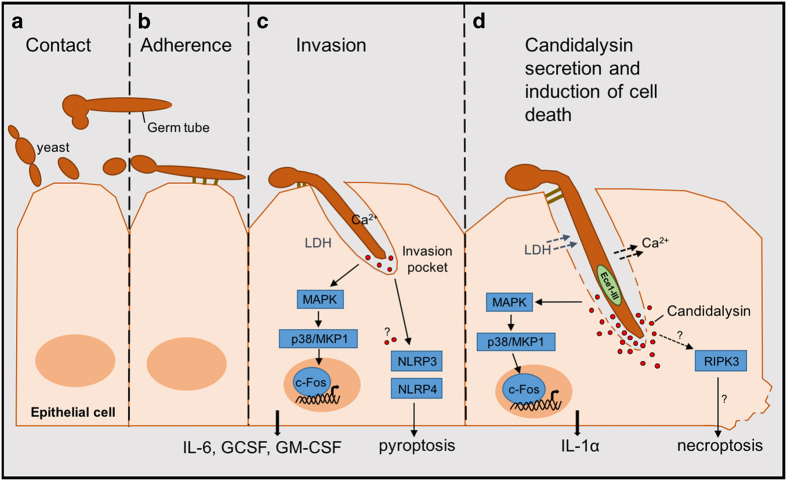
(**a**) *C. albicans* yeast cell makes contact with the host epithelium, forming germ tubes (the precursors of hyphae). (**b**) Germ tubes/hyphae mediate the initial adherence and invasion process via the fungal adhesin, Hwp1, and invasins, Als3 and Ssa1. (**c**) Invasion of host epithelial cells via hyphal extension, followed by early toxin secretion into the invasion pocket. Immune signaling events are triggered and immune cytokines produced. Hyphal morphogenesis induces pyroptosis, although it is uncertain whether this is indirectly or directly mediated by Candidalysin. (**d**) Late toxin secretion leading to membrane damage, lactate dehydrogenase release and calcium influx. Immune activation continues along with release of the damage-associated cytokine, IL-1*α*. Non-pyroptotic cell death ensues; however, questions remain as to whether Candidalysin release mediates alternative mechanisms of cell death such as necroptosis.
